# *In vitro* effect of hydroxyethyl starch on COVID-19 patients–associated hypofibrinolytic state

**DOI:** 10.1016/j.rpth.2024.102382

**Published:** 2024-03-15

**Authors:** Hamdi Rezigue, Michel Hanss, Jean-Stéphane David, Yesim Dargaud, Christophe Nougier

**Affiliations:** 1 Laboratoire d'hématologie, Groupement Hospitalier Est, Hospices Civils de Lyon, Lyon, France; 2 UR4609 Hémostase & Thrombose, Université Claude Bernard Lyon 1, Lyon, France; 3 Service d'anesthésie réanimation, Centre Hospitalier Lyon Sud, Hospices Civils de Lyon, Pierre-Bénite, France

**Keywords:** COVID-19, fibrinolysis, fibrin polymerization, hydroxy ethyl starch, thromboelastometry

## Abstract

**Background:**

Despite systematic thromboprophylaxis, 30% of the COVID-19 patients in intensive care units develop thrombosis. This occurrence is associated with a hypofibrinolytic state measured by thromboelastometry when adding tissue plasminogen activator (tPA) to citrated whole blood for a further run for EXTEM (ROTEM).

**Objectives:**

Because hydroxyethyl starches (HESs) affect fibrin polymerization, we have assessed its potential effect on *in vitro* tPA-induced fibrinolysis.

**Methods:**

Fifteen successive COVID-19 patients from the local intensive care units were selected for tPA resistance occurrence. HES was added to whole blood samples with proportion similar to the pharmacologic recommendations. Samples were run for EXTEM on a ROTEM delta device after further addition of tPA. Paired controls were whole blood samples with the same volume of saline added. To assess the impact of HES on coagulation, thrombin generation was measured in 10 COVID-19 patients in the presence of either HES or saline; then, the clots obtained were used to generate electron microscope images.

**Results:**

Clot firmness at 5 minutes and the lysis index at 30 minutes were decreased in presence of HES compared with saline (Wilcoxon test, *P* < .01 for HES vs saline and HES vs untreated). However, no statistically significant difference was observed for all thrombin generation assay parameters studied (endogenous thrombin potential, peak thrombin, and time to peak). With HES, fibrin fibers of either COVID-19 patients or control subjects were thicker than those of saline-treated samples.

**Conclusion:**

These results highlight that HES increased apparent *in vitro* tPA-induced fibrinolysis in case of severe COVID-19 disease. Use of this plasma volume expander may translate as a potential help against COVID-19–induced thrombosis occurrence.

## Essentials

• Thromboelastometry is relevant to screen hypofibrinolytic states.
• Hydroxyethyl starch (HES) appears to enhance *in vitro* fibrinolysis in COVID-19 patients.
• HES may have an influence on fibrin fiber diameter in COVID-19 patients.
• HES could be evaluated as a supportive therapy with respect to contraindications.

## 1 Introduction

Despite systematic thromboprophylaxis, 30% of COVID-19 patients in intensive care units could develop thrombosis [1]. This high prevalence is associated with a hypofibrinolytic state detectable by thromboelastometry (ROTEM) by lysis index at 30 minutes (Ly30) when adding recombinant tissue plasminogen activator (rtPA) to EXTEM in citrated whole blood [2]. The observed COVID-19–associated hypofibrinolysis is independent of the main plasma fibrinolytic components and is mostly related to a fibrin defect [3]. Bachler et al. [4] and Weiss et al. [2] previously demonstrated that critically ill COVID-19 patients experiencing a thrombosis were resistant to *in vitro* fibrinolysis initiated with rtPA in ROTEM [2].

Hydroxyethyl starch (HES) solutions were used for decades for plasma volume replacement following acute blood loss [5]. Recently, they were suspended across the European Union because of the risk of kidney injury and death in certain patients. HESs are polysaccharides produced from natural starch molecules extracted from corn or potatoes. These starch molecules are hydroxylated to slow down the *in vivo* hydrolysis of the macromolecule by alpha amylase [5].

It has been well described that HES can have an impact on coagulation mechanisms. Decrease in von Willebrand factor [6], factor VIII [6], and platelet function disorders [7] may be associated with the administration of HES. However, little is known about the effect of HES on fibrin formation and degradation.

As HES affects fibrin polymerization [8], the goal of the present study was to search for the *in vitro* effect of HES on tissue plasminogen activator (tPA)-induced fibrinolysis in critically ill COVID-19 patients with objectively determined hypofibrinolytic state.

## 2 Methods

### 2.1 Tissue plasminogen activator-thromboelastometry analysis

Adult patients with a positive COVID-19 polymerase chain reaction test hospitalized in Lyon University Hospitals were included. The study was approved by the institutional ethics committee (CPP Est IV 20/41 [COVID]/SI 20.04.23.41107), and informed consent was obtained from all participants or their relatives. Fifteen critically ill COVID-19 patients with hypofibrinolytic state, as objectively detected with tissue plasminogen activator-thromboelastometry (tPA-TEM) assay, were included in the study.

Clot formation and fibrinolysis were recorded using a ROTEM Delta device (Werfen) and EXTEM reagent in the presence of tPA 0.625 μg/mL (Actilyse, Boehringer-Ingelheim). Normal values were previously determined in the control population, which consisted of 30 healthy adult volunteers (14 men and 16 women) aged 22 to 58 years (mean ± SD, 32.4 ± 13.8 years), nonsmokers, not taking any medication known to affect the coagulation system, and with no history of venous thromboembolism or coagulation disorder.

HES was added to whole blood samples in a proportion similar to clinical recommendations (28/100 vol/vol) [9]. Matched controls were whole blood samples added with the same volume of saline. The amplitude at 5 minutes (A5) and the Ly30 were compared in the presence of HES and saline.

### 2.2 Thrombin generation assays

To evaluate the impact of HES on coagulation, thrombin generation was measured in same COVID-19 patients (*n* = 10) in the presence of either HES or saline. Briefly, 80 μL of platelet-poor plasma was dispensed into the round-bottom wells of 96-well microtitre plates (Greiner). A total of 20 μL of a mixture containing tissue factor and phospholipids (PPP reagent, Stago) was added to the plasma sample. Another 20 μL of Heptem reagent (Tem Innovation) containing heparinase was added to each well to neutralize the heparin in the patient’s plasma. Then, 36 μL of HES or saline was added, and the starting reagent (20 μL per well) contained fluorogenic substrate and CaCl_2_. Thrombin generation was measured in a Fluoroscan Ascent fluorometer (Thermolabsystems) using the calibrated automated thrombin generation assay (TGA), and a dedicated software program, Thrombinoscope (Diagnostica Stago), enabled the calculation of thrombin activity and displayed thrombin activity with time. All experiments were carried out in duplicate. Endogenous thrombin potential (area under the thrombin generation curve), peak thrombin height, and time to peak were analyzed.

### 2.3 Scanning electron microscopy

TGAs were carried out as described above but adapted for a plasma volume of 20 μL. TGAs were performed in 12 patients (6 COVID-19 and 6 septic patients free of COVID-19). After the thrombin generation reaction, clots obtained were fixed with 1.5% glutaraldehyde (Sigma) in phosphate-buffered saline. They were washed with phosphate-buffered saline and then fixed with 1% osmium tetroxide in sodium cacodylate. Then, they were dehydrated in ethanol, stored under vacuum, and placed on carbon adhesive strips. The surfaces of the samples were coated with gold-palladium ions and observed with a Hitachi S800 FEG scanning electron microscope (Hitachi). The diameters of 250 randomly selected fibrin fibers were measured in each sample using ImageJ software (National Institutes of Health).

### 2.4 Statistical analysis

Statistical analysis was performed using Prism 8 software (GraphPad Software Inc). Thrombin generation data were expressed as median (IQR). Electronic microscopy data were expressed as mean ± SD. Data were compared using the Wilcoxon signed-rank test. A *P* value of <.05 was considered statistically significant.

## 3 Results and Discussion

### 3.1 Effects of addition of HES on tPA-TEM analysis

We observed that the addition of HES partially restored fibrinolysis while the addition of saline solution did not (Figure 1A, B). In the presence of HES, we observed a significant decrease of both the amplitude at 5 minutes (A5 and Ly30) (Figure 1A, B). An inhibitory effect of HES on coagulation and an enhancing effect on fibrinolysis in tPA-resistant COVID-19 patients was suspected.

**Figure:**
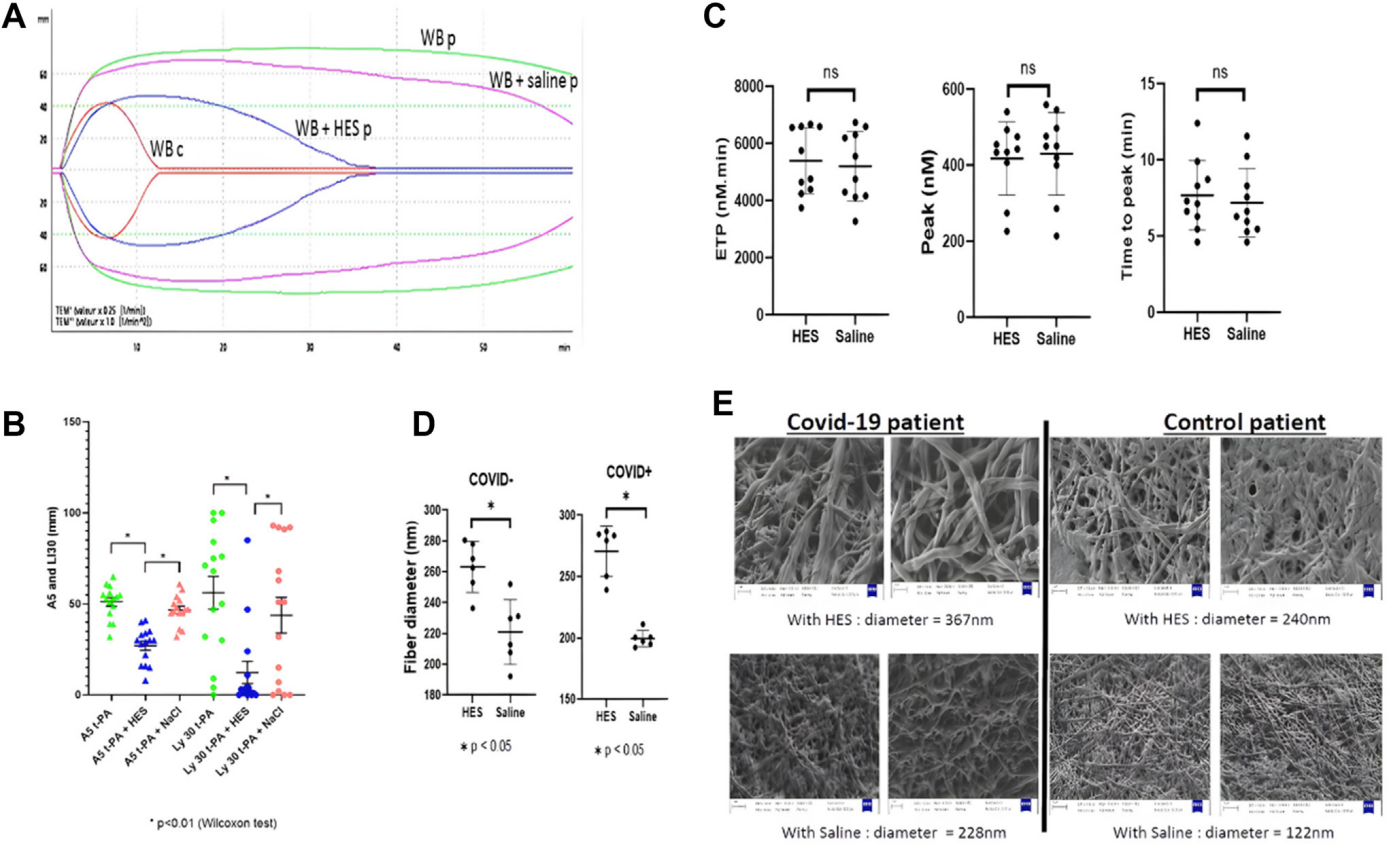
*In vitro* effect of hydroxyethyl starches (HESs) on blood samples of COVID-19 patients with hypofibrinolysis. (A) tissue plasminogen activator-thromboelastometry (tPA-TEM) curves obtained from a single patient with fibrinolysis resistance (WBp) and control patient (WBc). The green TEM-tPA curve represents a COVID-19 patient with a fibrinolysis resistance profile. The pink curve is the same patient with the addition of NaCl, and the blue curve is with the addition of HES. The red curve is a normal tPA-TEM control. HES seems to have an inhibitory effect on coagulation and an enhancing effect on fibrinolysis in tPA-resistant COVID-19 patients. (B) Comparison of the amplitude at 5 minutes (A5) and the amplitude of lysis at 30 minutes (Ly30) in the presence of HES and saline in tPA-resistant COVID-19 patients. Green represents patients with resistance to fibrinolysis, blue represents the same patients with HES treatment, and pink represents the same patients treated with NaCl. Clot firmness at 5 minutes was decreased in presence of HES compared with saline and untreated samples. Comparable differences were obtained at 30 minutes. (C) Comparison of endogenous thrombin potential, peak thrombin, and time to peak. No statistically significant difference was observed for all TGA parameters studied. (D) Comparison of fibrin filament diameters in the presence of HES and NaCl in non-COVID-19 and COVID-19 patients. In the presence of HES, fibrin fibers of COVID-19 patients were thicker compared with saline-treated samples. Similar effect of HES on fibrin was also observed in control subjects. (E) Electronic microscopy images obtained from a COVID-19 patient and a non-COVID-19 patient. With HES, fibrin fibers seem to be thicker compared with saline.

### 3.2 Effects of addition of HES on thrombin generation

To assess the impact of HES on coagulation, we compared HES-spiked plasma with saline-spiked plasma.

We found that the endogenous thrombin potential, the thrombin peak, and the time to peak were not modified by the addition of HES (5253 nM/min [4352-6592 nM/min], 438 nM [373-479 nM]), and 7.2 minutes [6.1-9.0 minutes], respectively) or saline solution (5149 nM/min [4152-6381 nM/min], 450 nM [371-509 nM], and 6.4 minutes [5.4-8.8 minutes], respectively).

No statistically significant difference was observed for all TGA parameters studied (endogenous thrombin potential, peak thrombin, and time to peak; Figure 1C). It would therefore appear that HES does not affect thrombin generation in platelet-poor plasma, while it seems to have an inhibitory effect on fibrinolysis measured in whole blood by ROTEM.

We hypothesized that HES could influence fibrin polymerization. As a result, tPA might be therefore more effective on the abnormal, more fragile fibrin fibers that may restore fibrinolytic activity in tPA-resistant COVID-19 patients. Same results have been observed in healthy plasmas (data not shown).

### 3.3 Effects of addition of HES on fibrin fibers with electronic microscopy

To assess the impact of HES on fibrin network, fibrin ultrastructure was studied using scanning electron microscopy. Hyperfibrinolysis can have an impact on clot structure (particularly in the case of COVID-19 infection). In our study, we compared plasmas with identical fibrinogen concentrations spiked with either HES or saline. It has been previously shown that thin fibrin fibers are more resistant toward fibrinolysis, while a fibrin network mostly formed by thicker fibrin fibers is looser and easily degradable by fibrinolysis [10]. In the presence of HES, fibrin fibers of COVID-19 patients were thicker (mean ± SD, 270.7 ± 20.5 nm) compared with those of saline-treated samples (199.5 ± 6.6 nm). Similar effect of HES on fibrin was also observed in control subjects. The mean fibrin fiber diameter was 263.2 ± 16.6 nm in the presence of HES and 221.0 ± 21.0 nm in the presence of saline solution. These results are in accordance with a previous publication showing that HES at concentrations as low as 2.5 mg/mL shortened the lag phase of turbidity increase and caused increased fibrin fiber mass/length ratios in both purified fibrin and plasma systems [8].

These data suggest that HES may have an influence on fibrin fiber diameter. It is well known that fibrin fiber diameter is dependent on the amount of thrombin-inducing fibrin polymerization; however, in our patients, we did not observe any effect of HES on thrombin generation.

## 4 Conclusion

These results indicate that HES, a therapeutic molecule known to affect fibrin polymerization, appears to enhance *in vitro* fibrinolysis induced by tPA in patients with severe COVID-19 infection presenting a hypofibrinolytic state and thrombosis. However, hypofibrinolysis can be observed in other pathologic situations, such as in traumatology, where fibrinolytic shut-down is well described. In this sense, TEM-tPA could be of interest if it was possible to screen polytrauma patients with a state of hypofibrinolysis associated with or without thromboembolic events. The tPA-TEM screening test would therefore enable individualized therapeutic adaptation as well as potential biological monitoring. In these patients, HES could also be tested to assess the restoration of fibrinolysis.

Our electron microscopy results confirm abnormal fibrin ultrastructure, with thicker fibrin fibers and a looser fibrin network in critically ill COVID-19 patients with thrombosis. In cases where venous or arterial thromboses occurred despite effective heparin prophylaxis in patients with severe SARS-CoV-2 infection, the supportive use of HES in combination with anticoagulant therapy might be of interest, in particular in patients developing massive pulmonary embolism (PE), which is responsible for high mortality. Endothelial cells of lungs have a high expression of plasminogen activator inhibitor 1) [11], which is further increased in patients with severe COVID-19 infection [12]. In these patients, a supportive HES therapy that may improve fibrinolytic activity in lung vessels might be of clinical interest. During COVID-19 pandemic, clinical trials evaluating the effect of systemic or catheter-delivered thrombolysis were carried out in critically ill COVID-19 patients with PE [13]. Despite clinical improvement in some patients, the overall mortality remained very high, almost 90% [13]. The failure of systemic or catheter-directed rtPA to improve SARS-CoV-2–associated PE is in accordance with tPA resistance observed in these patients. In addition, thrombolytic agents were found to be associated with higher risk of major bleeding and intracranial hemorrhage compared with anticoagulants (9.24% vs 3.42% and 1.46% vs 0.19%, respectively), which limits their clinical use [14]. However, in patients with severe, life-threatening COVID-19–associated PE, treatment options are limited and the use of HES might be considered. The bleeding risk associated with HES in this particular situation needs to be studied. *In vitro* data reported here open perspectives for a clinical trial evaluating the efficacy and safety of HES as a supportive therapy in critically ill COVID-19 patients with PE with respect to contraindications.
